# Natriuretic Peptides: The Case of Prostate Cancer

**DOI:** 10.3390/molecules22101680

**Published:** 2017-10-10

**Authors:** Letizia Mezzasoma, Matthew J. Peirce, Alba Minelli, Ilaria Bellezza

**Affiliations:** Dipartimento di Medicina Sperimentale, Università di Perugia, 06123 Perugia, Italy; letizia.mezzasoma@unipg.it (L.M.); mattpeirce69@gmail.com (M.J.P.); alba.minelli@unipg.it (A.M.)

**Keywords:** cardiac hormones, ANP, BNP, inflammation, cancer therapy

## Abstract

Cardiac natriuretic peptides have long been known to act as main players in the homeostatic control of blood pressure, salt and water balance. However, in the last few decades, new properties have been ascribed to these hormones. A systematic review of English articles using MEDLINE Search terms included prostate cancer, inflammation, cardiac hormones, atrial natriuretic peptide, and brain natriuretic peptide. Most recent publications were selected. Natriuretic peptides are strongly connected to the immune system, whose two branches, innate and adaptive, are finely tuned and organized to kill invaders and repair injured tissues. These peptides control the immune response and act as anti-inflammatory and immune-modulatory agents. In addition, in cancers, natriuretic peptides have anti-proliferative effects by molecular mechanisms based on the inhibition/regulation of several pathways promoting cell proliferation and survival. Nowadays, it is accepted that chronic inflammation is a crucial player in prostate cancer development and progression. In this review, we summarize the current knowledge on the link between prostate cancer and inflammation and the potential use of natriuretic peptides as anti-inflammatory and anticancer agents.

## 1. Natriuretic Peptides: Background

**Figure 1:**
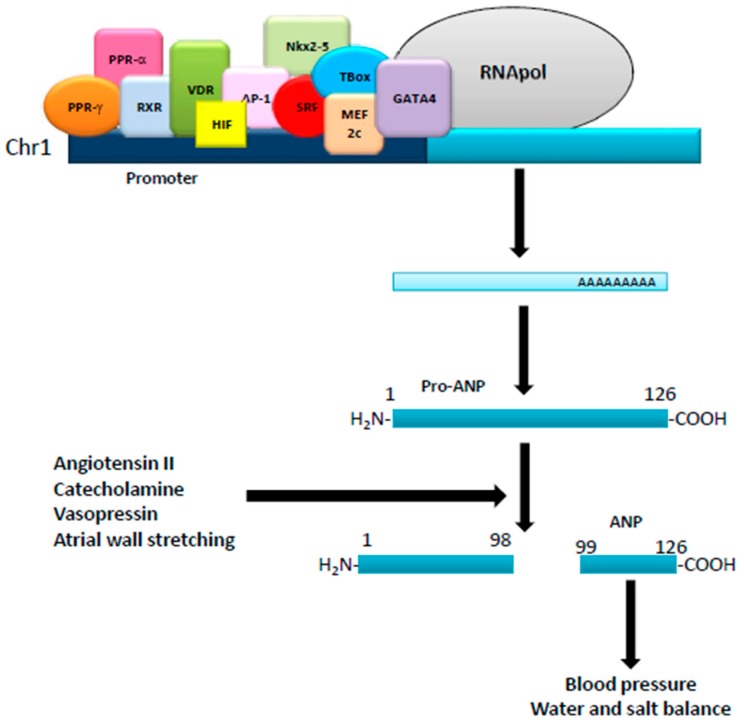
Regulation of ANP production. The human ANP gene, located in the short arm of chromosome 1, is regulated by several transcription factors acting on its promoter. Translated into a pro-ANP molecule of 126 aminoacids, it is then matured by different stimuli into the active ANP that exerts different physiological effects.

Natriuretic peptides, such as atrial (ANP), brain (BNP), and type-C (CNP), are a family of genetically distinct peptides with similar structure/function that share common membrane receptors [1]. CNP, produced and secreted from the vascular endothelium and male genital glands, and acts as a relaxing peptide, whereas ANP and BNP, mainly produced by atrial and ventricular myocytes [1], act as cardiac hormones. ANP originates from the cleavage of a 151 amino acid precursor (pre-proANP) that produces a 126 amino acid peptide (pro-ANP 1-126), stored in the atrium. Physical (atrial wall stretching) and/or hormonal (angiotensin II, catecholamines and vasopressin) stimuli initiate cleaveage of the pro-ANP1-126 into two fragments: a NH2-terminal (pro-ANP 1–98) and a COOH-terminal ANP 99–126 (ANP), the biologically active hormone [2,3,4]. ANP, composed of 28 amino acids, is characterized by a ringed structure due to intramolecular cysteine disulfide linkages. Kidney, vessels, and adrenal glands are the main targets of ANP, which, via different mechanisms, induces strong diuretic, natriuretic, and vasorelaxant effects, therefore acting as a major player in the homeostatic control of blood pressure, salt and water balance [2] (Figure 1). In humans, after i.v. injection, the half-life of ANP is 2–4 min: it can be degraded by metallo-endopeptidases as well as by binding to its clearance receptor, i.e., NPR-C. The human ANP gene is located on the short arm of chromosome 1 with a promoter region containing binding sites for many transcription factors, such as PPARγ, PPARα, retinoid-X-receptor (RXR), vitamin D receptor (VDR), hypoxia-inducible factor 1-alpha (HIF-1α), activator protein 1 (AP1), serum response factor (SRF), Nkx2-5, GATA binding protein 4 (GATA4), myocyte enhancer factor 2C (Mef2c) and T-Box factors [5]. ANP mRNA has been found in several organs and cell types, such as lung, brain, liver, gastrointestinal tract, thymus, vascular smooth cells, endothelial and immune cells, where it can be regulated by calcitonin gene-related peptide (CGRP), glucagon-like peptide-1 (GLP-1) as well as daily exercise and physical activity [4,6,7]. BNP is a 32 amino acid peptide, structurally similar to ANP [3]. The human BNP gene, located on chromosome 1, codifies a 132 amino acid peptide (pre-proBNP), cleaved and modified by corin and furin to a 108 amino acid peptide (proBNP) and stored as a mature hormone in the human heart [3]. When secreted by the heart, proBNP, besides post-translational modifications, is cleaved into a 32 amino acid peptide (the physiologically active hormone) and a 76 amino acid peptide (NT, a biologically inactive N-terminal fragment) [3]. BNP plasma half-life is 12–23 min, and its expression is controlled by adaptive mechanisms in response to myocardial stress. Its promoter region contains binding sites for several transcription factors, such as Yin Yang 1 (YY1), GATA-4, GATA-5, GATA-6, MEF-2, dHAND, SRF and Nkx2.5 [2,4]. Like ANP, physical activity upregulates BNP expression and release. CNP is a 22 amino acid peptide with a ring structure that lacks the carboxy-terminal extension [1]. The human gene (Nppc), located on chromosome 2, codifies a 126 amino acid peptide (pre-proCNP), which is cleaved into a 103 amino acid peptide (proCNP) and further processed by furin to produce the active hormone [3]. CNP plasma half-life is 2–3 min and its expression is controlled by cytokines and growth factors, such as tumor necrosis factor (TNF), lipopolysaccharide (LPS), basic fibroblast growth factor (bFGF), interleukin-1 (IL-1), transforming growth factor beta (TGF-β) and thrombin [8]. Its promoter region has binding sites for the transcription factor TSC-22 as well as NF-κB, STAT1, ATF6 and E2F1 [1,8].

ANPs bind to a specific plasma membrane receptor called natriuretic peptide receptor (NPR)-A, while BNP and CNP bind to NPR-B and NPR-C. NPR-A and NPR-B are transmembrane receptors coupled to an intracellular guanylate cyclase (GC) domain [9]. Their activation requires their hormone ligands that, upon binding to the extracellular domain of the receptor, cause a conformational change and the phosphorylation of six residues of the intracellular kinase homology domain (KHD). The subsequent hormone-induced receptor activation stimulates the GC domain, which, in turn, induces an intracellular production of c-GMP (cyclic-GMP) to activate a specific cGMP-dependent protein kinase (PKG) which stimulates several biological events [9]. On the other hand, NPR-C is a non-guanylyl cyclase receptor, coupled instead to the inhibitory guanine nucleotide regulatory protein (Gi). Thus, its activation leads to the inhibition of adenylyl cyclase or activation of PLC [10], with the homeostasis of the natriuretic peptides as its central biological effect. However, growing evidence suggests that the hormone can modulate cell growth, counteract oxidant-induced cell damage and inflammatory processes by influencing transcription of specific factors [2,11,12,13], thus connecting the physiological balance of the natriuretic peptides to a range of pathological conditions linked to these responses. For example, obesity, insulin resistance and diabetes, known to be linked both to chronic inflammation and increases in oxidative stress, are related to natriuretic peptides since patients with these pathologies all exhibit decreased levels of plasma/serum ANP and BNP. Moreover, this deficiency may contribute to enhancing their susceptibility to the risk of cardiovascular diseases [14,15,16].

## 2. Natriuretic Peptides and the Immune System

Under physiological/homeostatic conditions, immune responses depend on two interrelated systems: the innate and adaptive immune systems. Conventionally, monocytes, macrophages, granulocytes, neutrophils and dendritic cells (DC) are the major effectors of the innate immune system, while the adaptive immune response, characterized by its antigen specificity and memory, is mediated by lymphocytes. Innate immune system cells express pattern recognition receptors (PRR), that, by recognizing pathogen-associated molecular patterns (PAMPs) and damage-associated molecular patterns (DAMPs), trigger the production of endogenous signals, such as inflammatory cytokines/chemokines, thus alerting the host about danger. Moreover, antigen-presenting cells (APC), as sentinels alerted by PAMPs and DAMPs, perceive the existence of the antigen/neo-antigen, thus triggering the activation of the adaptive immune system. The development of an/immune response appropriate to the infectious (kill invaders) or non-infectious (repair injured tissues) insult depends upon a highly complex interplay between the innate and adaptive components of the immune system. Indeed, to avoid “collateral damage” to the host, all immune responses need to be finely tuned and organized such that the immune response lasts only as long as the insult itself [17]. Indeed, the failure of this fine-tuning, uncontrolled activation of the immune system or a failure to resolve after the initial challenge has been removed, is associated with chronic inflammation, which underpins a wide range of pathologies [18,19].

**Figure 2:**
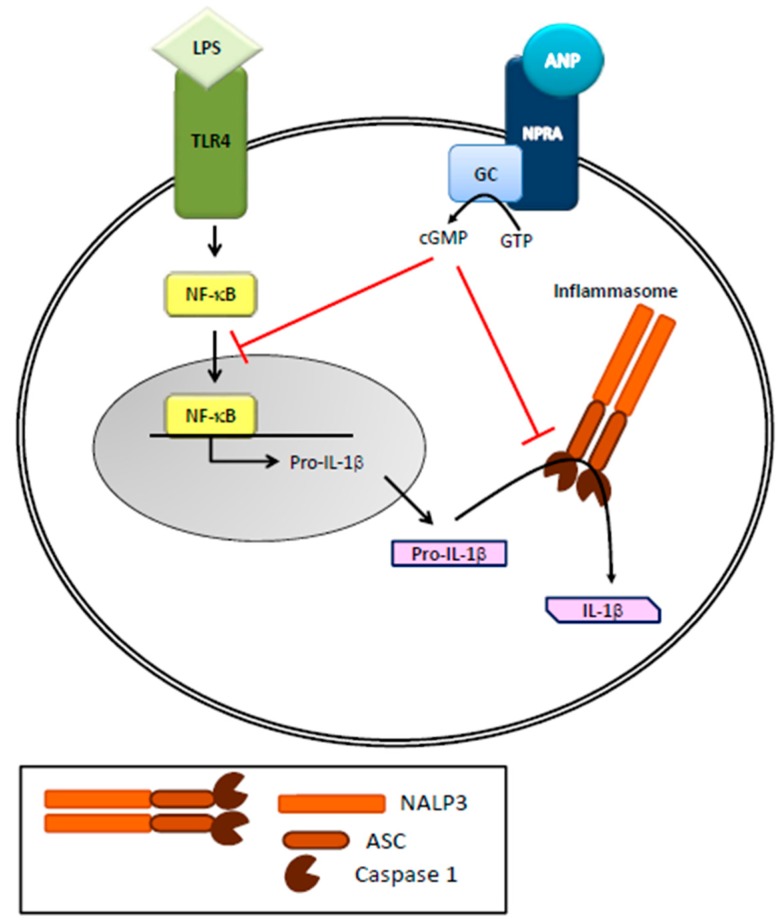
Role of ANP on inflammasome activation in immune cells. ANP, by binding to its receptor (NPRA), increases cGMP levels and inhibits LPS-induced IL-1β secretion in human THP-1 monocytes. LPS, through NF-κB activation, leads to the expression of pro-IL-1β that is then cleaved in to active IL-1β by NALP3 inflammasome/caspase-1 activation.

Toll-like receptors (TLRs) are key members of the PRR family expressed in innate immune cells, increase NF-κB transcriptional activity and play a critical role in controlling the development and character of adaptive immune responses. TLRs are linked to the activation of an array of signaling pathways including NF-κB and MAP kinases such as JNK, ERK and p38 that regulate expression of inflammatory cytokines such as TNF, inflammatory enzymes such as NOS, as well as inflammasome components that generate active IL-1β and IL-18. Together, these molecules provide the context for a developing adaptive immune response and direct its outcome. It is now accepted that ANPs are involved in innate immunity [2,20], since the cardiac hormones can stimulate superoxide anion production, leukotriene B4 synthesis and upregulation of CD11 expression in polymorphonuclear neutrophils (PMN) leading to the killing of pathogens. Neutral endopeptidases, mainly produced/released by PMNs, are involved in the degradation/clearance of ANPs, and their inhibition has therapeutic effects in heart failure [21]. Moreover, regulation of the immune and inflammatory response by ANPs is achieved via a tight control of macrophage functions and cytokine production. Indeed, immune ANP-producing cells, not only present specific natriuretic receptors, but are also regulated by inflammatory stimuli [12,22] that increase ROS and NO production [2]. More specifically, ANPs cause a strong stimulation of human neutrophil migration without deformation, enhance human natural killer cell cytotoxicity while inhibiting nitric oxide (NO) and tumor necrosis factor-α (TNF-α) production in murine macrophages [23,24,25]. Recently, Mezzasoma and colleagues (2016) [20] showed that the ANP/NPRA/cGMP axis downregulates LPS/ATP-induced IL-1β secretion in human THP-1 monocytes. Released by activated monocytes and macrophages, IL-1β is a very powerful inflammatory cytokine whose production is strictly controlled by NF-κB activation, which leads to the expression of pro-IL-1β. The precursor protein is then cleaved in to active/secreted IL-1β by NALP3 inflammasome/caspase-1 activation [26] (Figure 2). It is important to note that errant activation of NALP3 underlies the pathogenesis of a variety of human disease [27,28,29]. The anti-inflammatory and immune-modulatory effects of ANP, besides being related to NF-κB inhibition [24,30,31,32,33], are therefore linked to NALP3-inflammasome platform. Given that the ANP-dependent biological responses occur via its binding to NPRA, expressed in different type of immune cells [34], it has been reasonably suggested that, by autocrine/paracrine mechanisms [24,35], ANP exerts anti-inflammatory and immune-modulatory effects in the tissue microenvironment [20]. A similar biological effect based on similar molecular mechanisms, i.e., NF-κB and NALP3/ASC/Caspase-1 cascade inhibition, has been recently described for BNP [36], further supporting the anti-inflammatory role of this peptide [8,13,32,37,38,39,40,41]. As for direct effects on adaptive immunity, ANP/BNP reduce the number of CD4^+^CD8^+^ lymphocytes while increasing the CD4^−^ CD8^−^ cells and stimulate the differentiation of naïve CD4^+^ cells toward the Th2 and/or Th17 phenotype [42]. Moreover, CD4^(+)^LAP^(+)^ Treg frequency is negatively correlated with NT-proBNP concentration in patients with dilated cardiomyopathy [43,44]. In brief, data from the literature highlight a potential role of the natriuretic peptides to treat immune-related/ inflammatory diseases and IL-1β/NALP3-associated human disorders.

## 3. Natriuretic Peptides and Cancer

**Figure 3:**
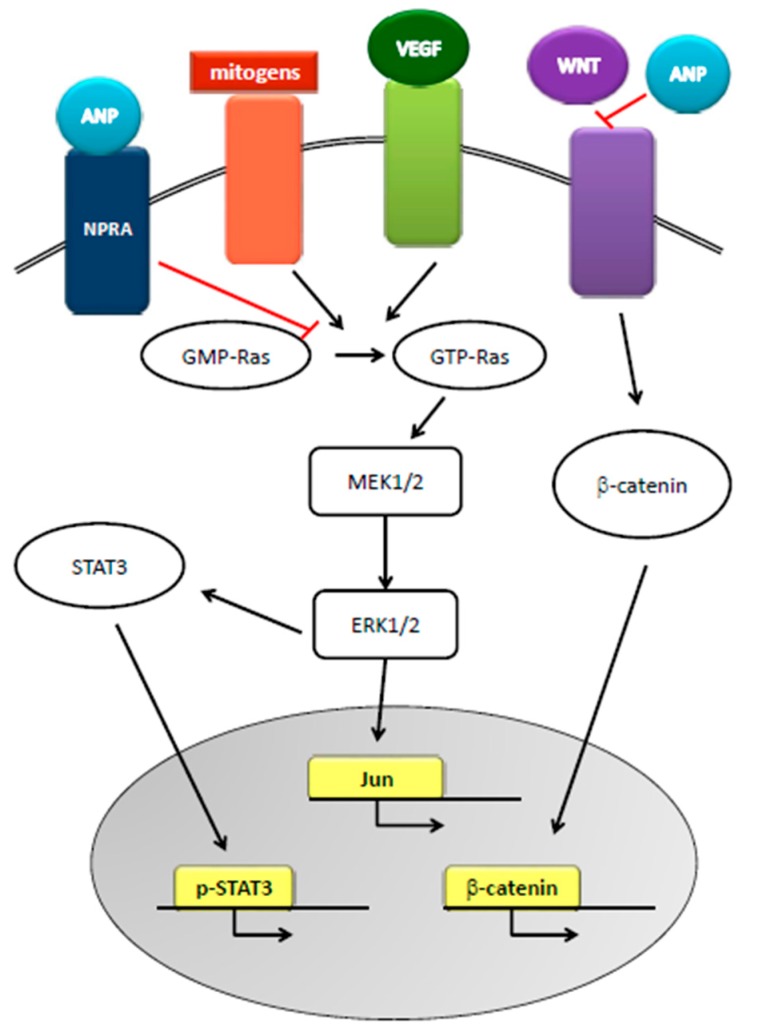
Role of ANP in cancer. ANP, by inhibiting the conversion of GDP-Ras to GTP-Ras, inhibits MEK 1/2-ERK 1/2 kinase cascade that is activated by mitogens and VEGF signaling. ANP also inhibits STAT3 activation and WNT-mediated β-catenin signaling, thus disrupting cellular cross-talks and inhibiting proto-oncogene actions.

The relationship between natriuretic peptides and cancer appeared in the late 1980s, when researchers reported the increased concentration of natriuretic factors in the presence of squamous cell carcinoma invasion, malignant pericardial effusion, and small cell lung cancer and tumor cell lines. To date, a thorough search in the literature resulted in 758 publications on the subject. Natriuretic peptides can inhibit the progression of pancreatic-, breast-, small cell lung-, and prostate cancer in vivo and in vitro [45] and have been proposed as primary treatments for cancers [46,47,48] since they cause the death of cancer cells without affecting healthy cells [29,49,50,51]. Mechanisms underlying the anti-cancer effects of natriuretic hormones are based on the inhibition of conversion of GDP-Ras to GTP-Ras, one of the main pathways in cancer formation, and on the cGMP-mediated inhibition of the Ras-MEK 1/2-ERK 1/2 kinase cascade. Furthermore, the hormones inhibit the crosstalk between Ras-MEK 1/2-ERK 1/2 kinase cascade and the pathways of VEGF, β-catenin, JNK, WNT, and STAT3, leading to the inhibition of the proto-oncogenes c-FOS and c-JUN in the nucleus of cancer cells [48,49] (Figure 3). Given that tumor progression and malignancy also require the preservation of extracellular acidosis, cancer cells overexpress on their membrane several pH regulators. ANP inhibits the amiloride-sensitive Na^+^/H^+^ exchanger isoform 1, thus disrupting cancer cell pH homeostasis and reducing cell survival [49,50,51]. However, some groups [38,52,53,54] reported that plasma BNP levels are elevated in patients with cancer and suggested that these increases are due to the cardiac response to cancer-related inflammation. Indeed, BNP is upregulated at the transcriptional and translational levels by pro-inflammatory cytokines in myocardiocytes [55] and patients with hematologic malignancies have elevated levels of N-terminal –proBNP, related to a possible myocardium response to the setting of the cancer [56,57]. Serum levels of N-terminal pro- C-type NP are associated with bone formation activity in patients with multiple myeloma [58], and act on mice lung fibroblasts to reduce pulmonary fibrosis [59]. CNP, in combination with sidenafil, inhibits rhabdomyosarcoma cells proliferation [60]. In general, the guanylin-hormone receptors, as cGMP inducers, can regulate tumor cell proliferation [61] and are now accepted as novel targets in inflammation, cancer, and cancer-related inflammation [32,62,63,64].

## 4. Prostate Cancer and Inflammation

Prostate cancer is one of the most common cancers in the male population, representing 20% of newly diagnosed malignancies in Italy in 2015 (AIRTUM) [65]. Whereas localized PCa can be treated with surgery or radiation, metastatic tumors do not clinically benefit from these treatments. The ultimate therapeutic option for such patients is the androgen deprivation therapy (ADT), which leads to an initial regression followed, in the vast majority of the cases, by a tumor relapse into castration resistant PCa (CRPC), a particularly aggressive phenotype for which there is currently no therapeutic treatment available [66,67,68]. Indeed, Docetaxel, the only approved drug for CRPC, enhances survival by approx. 2–3 months and new chemotherapeutic agents, such as cabazitaxel, abiraterone and enzalutamide, although slightly improving the survival, cause resistance mainly due to mutation in the androgen receptor (AR) gene [69,70,71,72,73]. Hanahan and Weinberg, examining the biological capacities that are acquired during the multistep development of human tumors, included inflammation as a novel hallmark of cancer, since tumor-associated inflammatory response can enhance tumorigenesis and progression [74]. Inflammation may stimulate carcinogenesis by causing DNA damage (genetic and epigenetic modulations), promoting cellular proliferation as well as angiogenesis [75,76,77,78]. Nevertheless, inflammation as a self-determinant risk-factor for PCa development has been a controversial issue [79,80,81,82,83,84,85,86,87,88], although the disorders in the prostate are mainly aging-related [84] with a concomitant increase in the susceptibility of the prostate tissue to injury/infection leading to inflammatory response [79,89,90,91,92]. Nowadays, it is accepted that chronic inflammation, besides underlying several malignancies, such as gastric, colon, esophageal and lung cancers, and hepatocellular carcinoma [93,94], is a crucial player in PCa development and progression to advanced metastatic disease [95]. It appears that chronic inflammation is the link between environmental stimuli and PCa occurrence. Indeed, age, ethnicity, and family history are the only acknowledged risk-factors for development of PCa and these genetic traits might, at least partially, explain the geographical variance in PCa incidence and mortality [79,81]. Moreover, proliferative inflammatory atrophy (PIA) areas in the prostate, regarded as primary sites of cancer and precursor of prostatic intraepithelial neoplastic (PIN) lesions [79,96], denote focal atrophic lesions containing acute/chronic inflammatory infiltrate [97,98,99,100]. Chronic inflammation, by dynamically modulating tumor microenvironment, controls PCa progression and metastasis via angiogenesis and epithelial mesenchymal transition (EMT) [94,98,101]. It has been reported [93] that the infiltration of macrophages and immune suppressor cells is positively associated with PCa progression, since the infiltrating lymphocytes and the tumor-microenvironment, by secreting large amounts of cytokines/chemokines, trigger a vicious circle, which drives cellular activities to cancer progression and aggressiveness [102,103,104,105]. In general, although prostate cancer, as well as each type of human cancers, is characterized by a multistep development, targeting the chronic inflammation branch could have potential beneficial effects for the treatment of this deadly neoplasia [62,106,107].

## 5. Natriuretic Peptides and Prostate Cancer

All the physiological effects of natriuretic peptide hormones are mediated by the interaction with the cell surface natriuretic peptide receptor A (NPRA; high affinity) and natriuretic peptide receptor C (NPRC; low affinity) [54]. ANP overexpression decreases NPRA levels in cells by a feedback inhibition. NPRA expression and signaling plays a crucial role in tumor growth [32] and deficiency of NPRA in mice (NPRA-KO) protects from lung, skin and ovarian cancer as well as from inflammation. The natriuretic hormone peptide, NHP73-102, [108], by blocking the expression of NPRA (iNPRA), exerts robust anti-inflammatory and antitumor effects [108]. In 2005, the existence of NPRA in prostate cancer cells was demonstrated for the first time. This finding was later confirmed by Wang and colleagues (2011) [50] who demonstrated that NPRA is highly expressed in human and mouse PCa cell lines and in advanced PCa tissues, but not in a normal prostate epithelial cell line or in benign prostate hyperplasia epithelial cell line. In addition, they found ANP in culture supernatants of PC3 and DU145 prostate cancer cell lines as well as in culture supernatants of WPMY, a stromal cell line. ANP was not found in supernatants of normal prostate epithelial nor LNCaP cell lines. Based on these results, the Authors proposed that stromal cell-derived ANP binds to NPRA, expressed by androgen-dependent cells in a paracrine manner, while androgen-independent cells signal in an autocrine manner since produce both ANP and NPRA. Therefore, the ANP-NPRA pathway appears to be crucial to the interaction between stroma and prostate cells in PCa pathogenesis, and might represent an effective therapeutic target. Indeed, TRAMP-C1 cells, injected into C57BL/6 mice, induced tumors only in mice that had not been treated with iNPRA, thus confirming that the block of NPRA expression is valuable for the treatment of PCa. The beneficial effect of NPRA blockage is also ascribed, at least in part, to the downregulation of macrophage migration inhibitory factor (MIF), which, in turn, regulates IL-6 levels in PCa cells [50,109]. A drawback of iNPRA therapy for PCa arises from the physiological role of NPRA in blood pressure regulation. However, by comparing blood pressure of NPRA-KO and TRAMP mice, no relationship between NPRA expression, blood pressure levels and PCa incidence were found. These findings are in agreement with studies in humans, showing no relationship between blood pressure and PCa [110]. Moreover, NPRA expression is positively correlated with Gleason score and pathological staging in androgen-independent PCa. Hence, NPRA has been proposed as a clinical prognostic marker and target for PCa. On the other hand, the expression of NPRC in prostate tumor tissue has been exploited to establish a new nanoagent for prostate cancer PET imaging by synthesizing an amphiphilic comb-like nanoparticle containing C-atrial natriuretic factor (CANF) for NPRC receptor targeting and 1,4,7,10-tetraazacyclododecane-1,4,7,10-tetraacetic acid (DOTA) chelator for high specific activity Cu-64 radiolabeling [111]. As for the therapeutic use of natriuretic peptides, Serafino and Pierimarchi [49] defined ANP as a magic bullet for cancer therapy because it can inhibit tumor growth both in vitro and in vivo [45]. Indeed, these hormones, injected subcutaneously for one month by osmotic pumps, are capable of inhibiting the growth of human cancers, thus making these peptides attractive candidates for fighting cancer [46,47,48,51]. The capability of targeting several pathways, pivotal for cell neoplastic transformation and solid tumor survival, supports the value of ANP for preventive and therapeutic strategies, also based on the fact that neither cytotoxicity nor a single side-effect is associated with subcutaneous infusion of these peptide hormones, which, by pharmacokinetic analysis, is the optimal method of administration [48,49,112].

## 6. Conclusions

The intimate relationship between chronic inflammation and prostate cancer tumorigenesis, progression and metastasis should be exploited in the design of new cancer therapies. At the same time, the strong anti-proliferative and anti-inflammatory properties of natriuretic peptides point to these cardiac hormones as potential therapeutic golden bullets, devoid of the cytotoxicity/side-effects that, for many chemotherapeutic agents, exact too high a cost in life-quality for limited prognostic improvement. In this context, we envisage that full engagement of the energy and financial muscle of the pharmaceutical industry to this question could yield novel therapeutic modalities with greatly improved efficacy and patient compliance.
